# Blood Epstein–Barr virus DNA does not predict outcome in advanced HIV-associated Hodgkin lymphoma

**DOI:** 10.1007/s12032-018-1099-2

**Published:** 2018-03-13

**Authors:** Ikram Ul-Haq, Alessia Dalla Pria, Elisa Suardi, David J. Pinato, Fieke Froeling, John Forni, Paul Randell, Mark Bower

**Affiliations:** 1 National Centre for HIV Malignancy, Chelsea and Westminster Hospital, 369 Fulham Road, London, SW10 9NH UK; 2 Department of Pathology, Imperial College School of Medicine, London, UK

**Keywords:** EBV, Hodgkin’s lymphoma, HIV, Prognosis, Survival, Outcome

## Abstract

In HIV-seronegative patients with advanced Hodgkin lymphoma (HL), Epstein–Barr virus (EBV) viraemia at diagnosis predicts a worse progression-free survival (PFS), independent of the International Prognostic Score. However, its role in HIV-associated HL is uncharacterised. We collected clinico-pathologic and treatment data from a prospective series of 44 HIV-associated HLs from 2000 to 2016. We evaluated circulating EBV DNA as a prognostic factor on uni- and multivariable analyses in relationship to the International Prognostic Index criteria. In 44 patients with HIV-associated HL, EBV was detected by in situ hybridisation in all diagnostic biopsies. Blood EBV DNA was detectable in 26 patients (59%) with a median of 600 copies/mL (range 0–161,000). EBV DNA was independent of CD4 cell count (*p* = 0.9) or HIV viral load (*p* = 0.6) and did not predict PFS (HR 1.6, 95% CI 0.39–6.7, *p* = 0.49). EBV DNA is not a prognostic trait in HIV-associated HL. Prognostication in HIV-associated HL should be solely based on the International Prognostic Index criteria.

## Introduction

Hodgkin lymphoma (HL) is a neoplasm arising from germinal centre or post-germinal centre B lymphocytes. It is characterised by the presence of malignant Hodgkin/Reed–Sternberg (HRS) cells in the backdrop of an inflammatory infiltrate. Studies have demonstrated an increased incidence of HL in immunosuppressed populations, in particular people living with HIV (PLWH) [1]. Studies have found the incidence of HL in the HIV-infected population to be 15–17 times higher than compared in the age- and gender-matched general population [2, 3]. Furthermore, the incidence of HL in PLWH has increased since the introduction of combination antiretroviral therapy (cART) [4]. HL in immunosuppressed populations is almost always associated with the gamma herpesvirus Epstein–Barr virus (EBV) [5, 6]. Although early studies suggested that HL was more aggressive in PLWH and carried a poor prognosis [7], more recent studies have found no difference in clinical outcomes when PLWH are treated with cART and the same chemotherapy regimens as the general population with HL [8].

The International Prognostic Score (IPS) is a widely used risk stratification tool for advanced HL that was created by the International Prognostic Factor Project on Advanced Hodgkin’s Disease in 1998 [9]. It comprises seven parameters that are independently associated with a poorer clinical outcome: serum albumin less than 40 g/L, haemoglobin less than 105 g/L, male gender, age over 45 years, stage IV disease, white blood cell count ≥ 15,000/microL, and lymphocyte count less than 600/microL and/or less than 8 per cent of the white blood cell count.

A recent study in HIV-seronegative patients with advanced stage HL found that blood EBV DNA also predicted clinical outcomes [10]. Moreover, patients with detectable blood EBV DNA at HL diagnosis had a worse disease-free survival, independent of the IPS [10]. In this study, we examine the plasma EBV DNA detectability in HIV-positive patients with advanced HL and evaluate its prognostic value.

## Materials and methods

At the National Centre for HIV malignancies at the Chelsea and Westminster Hospital, we prospectively collect routine data on all individuals who attend including all HIV-seropositive patients diagnosed with lymphoma since 1986. Data on patient characteristics, prognostic factors, treatment and outcomes for patients diagnosed with advanced HL since 2000 was extracted from the database. The study complied with local regulations and was approved by the institutional review boards of Chelsea and Westminster Hospital. Patient details were anonymised, and therefore, patient consent was not required.

Quantitative EBV DNA testing methodologies evolved during the study period. The current standard is DNA extraction from whole blood samples **(**MagNA Pure LC 2.0, Roche) followed by PCR (Rotor-Gene Q, Qiagen) using a commercial assay (Artus EBV RG, Qiagen) with a limit of detection of 500 copies/mL. The EBV status was determined as detected or not detected according to the assay available at the time of sampling.

Comparison of variables between the groups was by Chi-square test for categorical data and Mann–Whitney or Kruskal–Wallis test for nonparametric continuous variables; all p values are two-sided. The time interval from HL diagnosis until death, study censoring or loss to follow-up was used to calculate overall survival. Progression-free survival was defined as the period of time from HL diagnosis to progression, loss to follow-up or death. Survival curves were plotted according to the method of Kaplan and Meier [11]. The log rank method was used to test for the significance of differences in survival distributions [12].

## Results

During the 16-year inclusion period (2000–2016), 44 PLWH and advanced HL were identified. The mean age at advanced HL diagnosis was 42 years (range 21–69), 40 (91%) were male, and 79% were established on cART of whom 88% had undetectable HIV viral load in plasma. The median CD4 cell count was 169/mm^3^ (15-782), and 84% had an IPS > 2.

All diagnoses were confirmed by central review of histopathology, and in situ hybridisation of Epstein–Barr virus-encoded small RNAs (EBERs) found that EBV was present in 100% of diagnostic tissue. However, blood EBV DNA at HL diagnosis was detectable in 26 patients (59%) and the median EBV DNA at diagnosis was 600 copies/mL (range 0–161,000). Detectable blood EBV DNA was not associated with the CD4 cell count (*p* = 0.9) or detectable plasma HIV viral load (*p* = 0.6).

One patient, who had advanced AIDS and active opportunistic infection (OI) at the time of HL diagnosis, was treated with best supportive care only. The remaining 43 patients were treated with combination chemotherapy with curative intent: 41 ABVD (doxorubicin, bleomycin, vinblastine, dacarbazine), 1 VEPEB (vinblastine, cyclophosphamide, procarbazine, prednisolone, mitoxantrone, bleomycin, etoposide) and 1 ChlVPP (chlorambucil, vinblastine, procarbazine, prednisolone). One of these 43 patients died in remission of OI and 10 relapsed. All 10 patients with relapsed HL were treated with salvage cisplatin-based chemotherapy. Two had chemorefractory disease, and both died after further lines of salvage chemotherapy. All of the remaining 8 patients with relapsed HL are alive in remission, including 6 who received high-dose chemotherapy and autologous stem cell rescue. Thus, in total 4 patients have died, 2 from chemorefractory relapsed HL, 1 from untreated HL with concomitant OI and 1 from OI in first remission of HL.

**Fig. 1:**
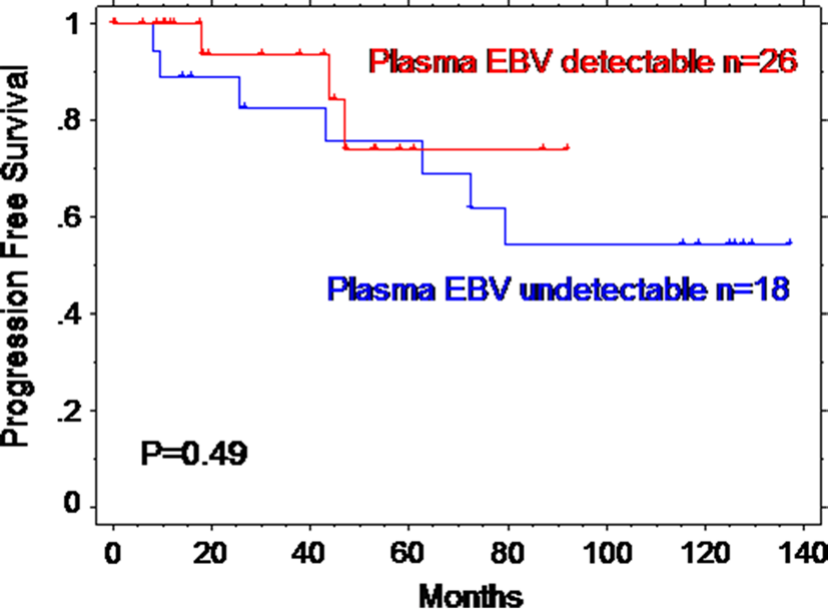
Kaplan–Meier curve of progression-free survival according to the presence of blood EBV at HL diagnosis

The median follow-up is 4.5 years (maximum 14 years). The 5-year overall survival (OS) for the 44 patients is 95% (95% confidence interval 88–100%), and the 5-year progression-free survival (PFS) is 76% (95% CI 6-092%). Detectable EBV did not correlate with any of the seven prognostic parameters that make up the IPS apart from anaemia (Hb < 105 g/L, *p* = 0.02, Table 1). In univariate analysis, detectable blood EBV viraemia was not associated with progression-free survival (hazard ratio 1.6, 95% CI 0.39–6.7 log rank *p* = 0.49, Fig. 1).

**Table 1:** Details of International Prognostic Score for the cohort of 44 patients with advanced HIV-associated HL and according to blood EBV status

IPS component	All	Detectable blood EBV	Undetectable blood EBV	*χ*^2^ test
Male	40/44	14/26	16/18	*P* = 0.7
Age > 45 years	28/44	15/26	13/18	*P* = 0.3
Stage IV	36/44	23/26	13/18	*P* = 0.2
Albumin < 40 g/L	42/42^a^	26/26	16/16	*P* = 1
Anaemia Hb < 105 g/L	23/42^a^	18/26	5/16	*P* = 0.02
Leucocytosis WCC > 15x10^9^/L	0/42^a^	0/26	0/16	*P* = 1
Lymphopenia < 0.6x10^9^/L	15/42^a^	10/26	5/16	*P* = 0.2
IPS 2^b^	7 (18%)	3 (12%)	4 (25%)	*P* = 0.3
IPS 3^b^	11 (28%)	6 (25%)	5 (31%)	
IPS 4^b^	7 (18%)	3 (12%)	4 (25%)	
IPS 5^b^	12 (30%)	9 (38%)	3 (19%)	
IPS 6^b^	3 (7%)	3 (12%)	0 (0%)	

^a^Values missing for 2 patients

^b^IPSs at diagnosis missing for 4 patients

## Discussion

The pathogenetic link between EBV and HL was suggested by epidemiological studies in the late twentieth century [13], and this was confirmed by a large Scandinavian population-based cohort study of young adults with infectious mononucleosis [14]. An elevated risk of HL was also demonstrated in immunosuppressed individuals, including allograft recipients [15] and PLWH [1, 16]. Moreover, HL in the context of immunosuppression is almost always associated with EBV infection [17, 18]. In these cases, EBV is present in the tumour cells of HL where the virus expresses a limited repertoire of latent genes including latent membrane proteins (LMP1 and LMP2a) and EBV-encoded small RNAs (EBERs) which can be detected by in situ hybridisation.

Quantitative PCR can measure the viral load of EBV in blood, and detectable blood EBV correlates closely with EBER-ISH in HIV-negative patients with advanced stage HL [10, 19, 20]. Analysis of samples from HIV-negative patients with advanced HL enrolled into a large North American Cancer Cooperative Intergroup Trial that enrolled 794 patients found EBER-ISH expression in only 51/315 (16%) of tissue samples available for analysis. Pre-treatment plasma samples were available for 274 patients, and 54 (20%) had detectable plasma EBV viraemia using a cut-off of 60 copies/100 µL. For 116 patients, both tissue for ISH and pre-treatment plasma samples were available and the concordance between tissue and plasma detection of EBV was very high at 96%. Despite the high concordance in these patients with both samples available, the prognostic significance of plasma EBV and tissue EBV-ISH differed. The HIV-negative study found that detectable EBV viraemia was associated with an inferior failure-free survival (*p* = 0.009), but tissue sample EBER-ISH did not predict outcome (*p* = 0.43). Moreover, pre-treatment detectable blood EBV was an independent prognostic variable after adjusting for IPS, treatment arm and histological subtype [10].

In contrast, in PLWH we found EBV by ISH in all 44 diagnostic tissue samples and detectable blood EBV in 59%. Because of the variation in the quantitative EBV DNA testing methodologies over the study period, direct comparison between the EBV viral load results was not undertaken and it was not possible to investigate the prognostic value of the level of the EBV DNA in the blood. However, analysis using detectable EBV viraemia as a nominal variable demonstrated that in PLWH, detectable blood EBV did not predict progression-free survival.

The reason for the discrepancy between these two findings is not clear. The sample size for the HIV-related advance HL is much smaller, and it is noteworthy that all tumour samples in PLWH had EBV detected by EBER-ISH, but this was only found in 16% of the HIV-negative series. Another discrepancy between the two studies is the concordance between tissue and plasma detection of EBV with a very high concordance in the HIV-negative study, whilst only 59% of PLWH had EBV viraemia, but all had EBV detected in HL tissue biopsies. Another possible confounder is the origin of the EBV viraemia. Previous studies have confirmed that EBV in HL tissues is latent rather than replicative in PLWH [17] and so the EBV viraemia may originate from replicative infection of other host cells. Under these circumstances, it might be expected that EBV viraemia would be higher in more immunosuppressed individuals, but there was no correlation between detectable blood EBV and CD4 cell count (*p* = 0.9) or detectable plasma HIV viraemia (*p* = 0.6).

## Conclusion

Although EBV is invariably detectable by in situ hybridisation in HL in PLWH, plasma EBV DNA was only detectable in 59% patients with advanced HL. In contrast to findings in HIV-negative patients with advanced HL, detectable blood EBV DNA did not predict overall or progression-free survival. Furthermore, it did not add to the prognostic value of the IPS.
